# Supplementary material for: Plasma Surface Modification of 3Y-TZP at Low and Atmospheric Pressures with Different Treatment Times
Source: Int J Mol Sci. 2023 Apr 21;24(8):7663. doi: 10.3390/ijms24087663 (PMC10144831; doi:10.3390/ijms24087663)
Supplement: Supplementary file 1 [file ijms-24-07663-s001.zip › Supplementary Figure annotation.docx]

Figure S1. XPS sputter depth profiling of the subsurface area of each specimen. From the nitrogen profiles, the zone of nitrogen diffusion at the surface may be up to ~17 nm thick depending on the plasma type and plasma exposure time.

Figure S2. (A) Rietveld refinement of the XRD data of “control” sample over the entire 2 theta rage measured. The circles indicate the data, the solid lines indicate the calculated model, the line at the bottom indicates the difference between the two, and the vertical bars indicate the position of the reflections. (B, C, D) Contributions of four different crystalline phases, tetragonal´, tetragonal, cubic and monoclinic, resolved by the Rietveld refinement in selected parts of the XRD data.

Figure S3. (A) Rietveld refinement of the XRD data of “A1” sample over the entire 2 theta rage measured. The circles indicate the data, the solid lines indicate the calculated model, the line at the bottom indicates the difference between the two, and the vertical bars indicate the position of the reflections. (B, C, D) Contributions of four different crystalline phases, tetragonal´, tetragonal, cubic and monoclinic, resolved by the Rietveld refinement in selected parts of the XRD data.

Figure S4. Various crystal structures of yttria-stabilized zirconia (YSZ) based on structural information from PDF database.
