# Supplementary figures and images for: Plasma Surface Modification of 3Y-TZP at Low and Atmospheric Pressures with Different Treatment Times
Source: Int J Mol Sci. 2023 Apr 21;24(8):7663. doi: 10.3390/ijms24087663 (PMC10144831; doi:10.3390/ijms24087663)

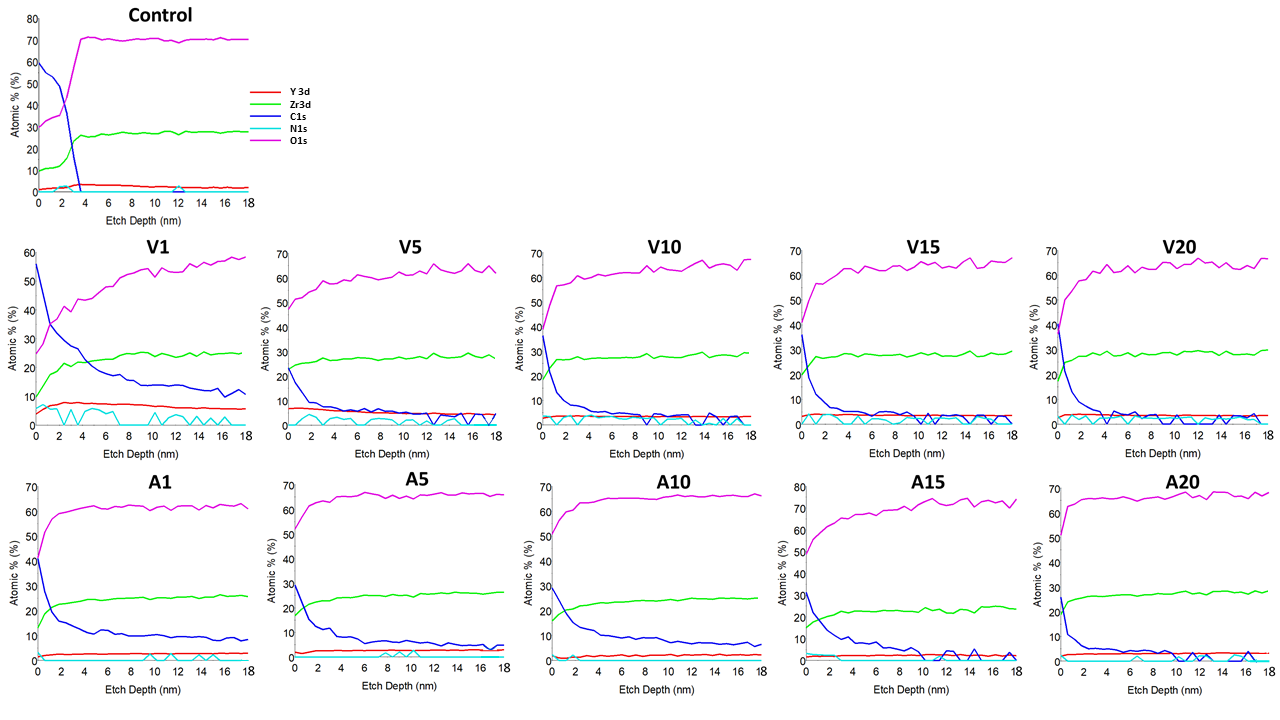

Supplement: Supplementary file 1 [file ijms-24-07663-s001.zip › Figure S1.tif]

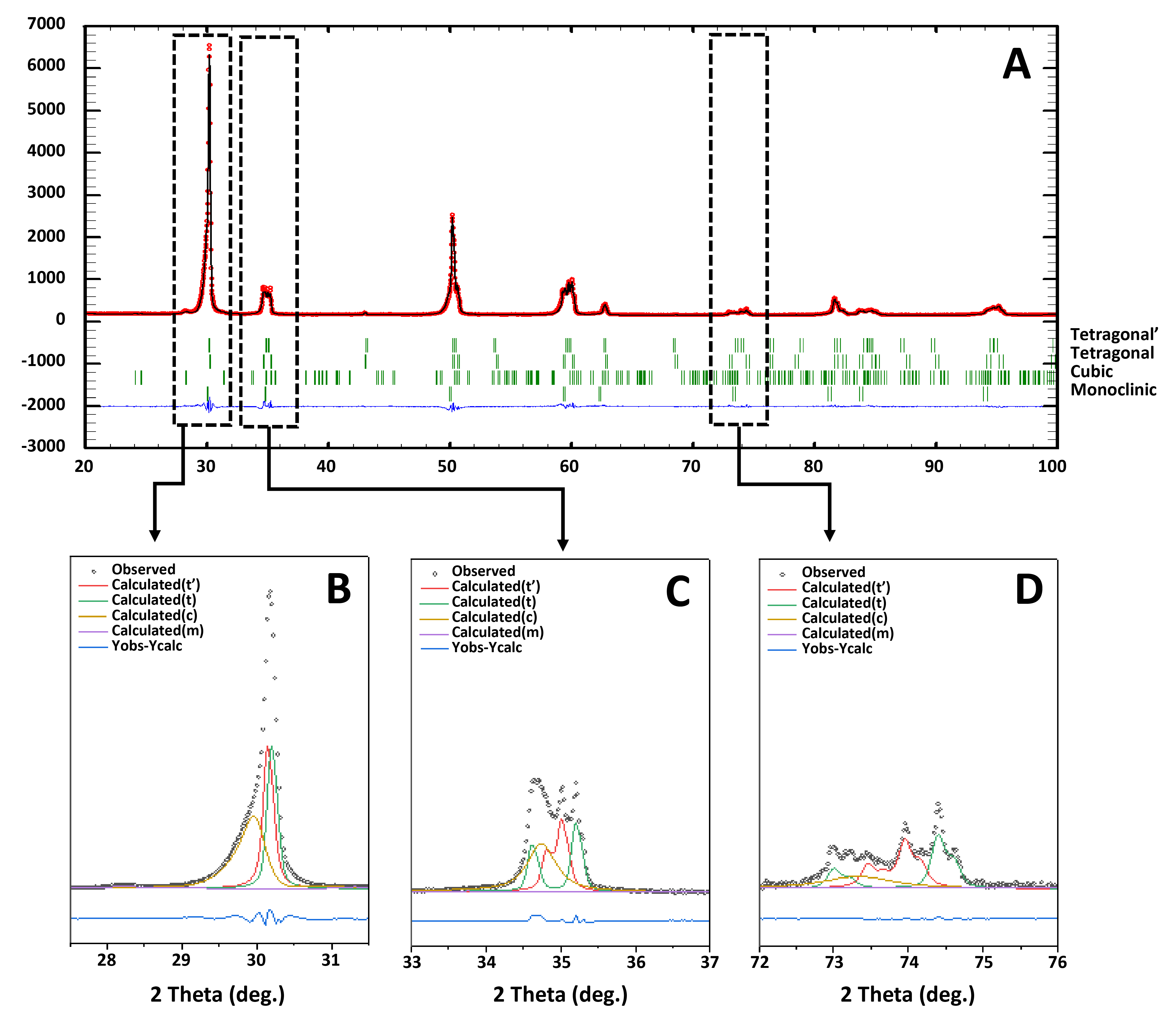

Supplement: Supplementary file 1 [file ijms-24-07663-s001.zip › Figure S2.tif]

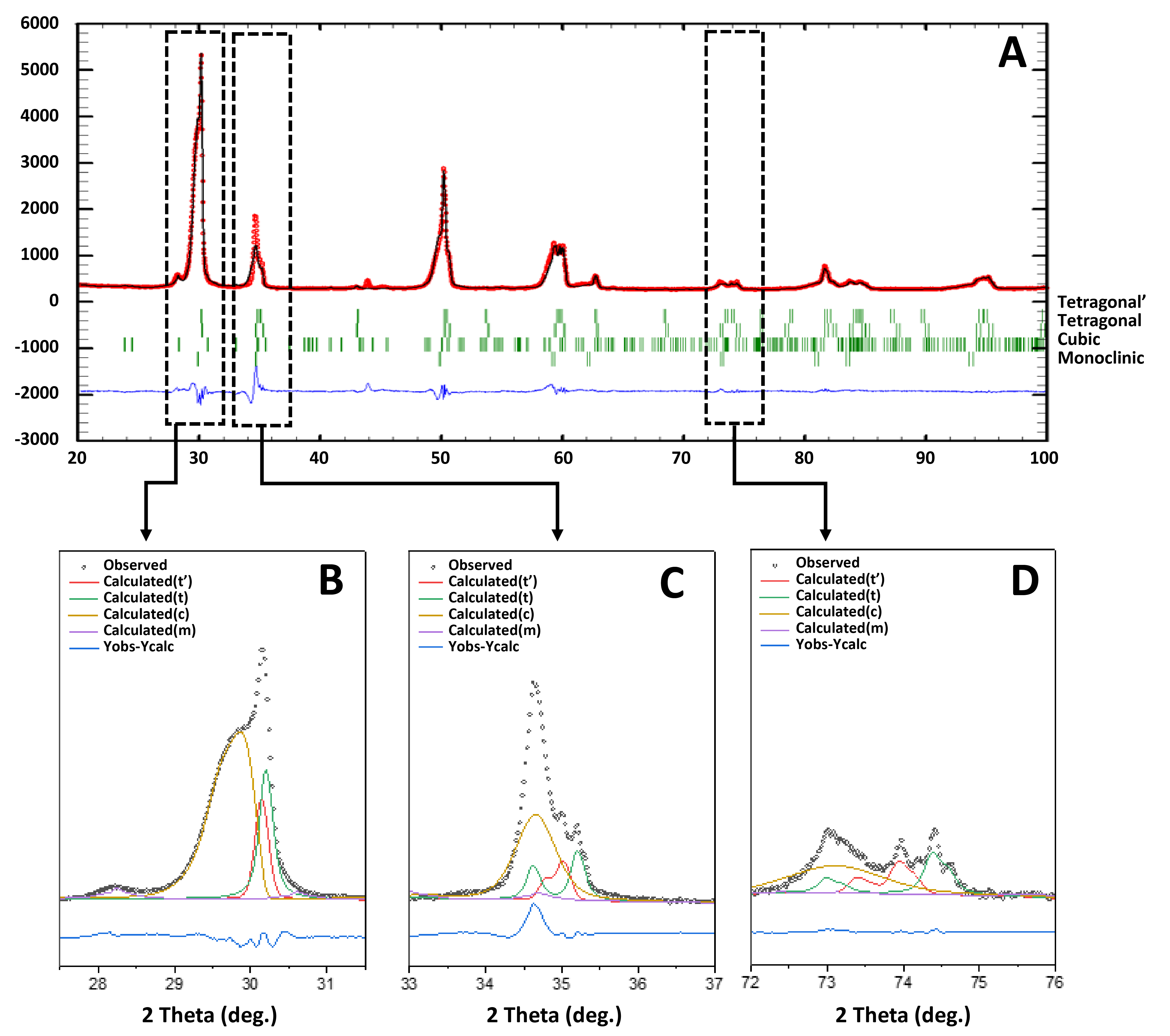

Supplement: Supplementary file 1 [file ijms-24-07663-s001.zip › Figure S3.tif]

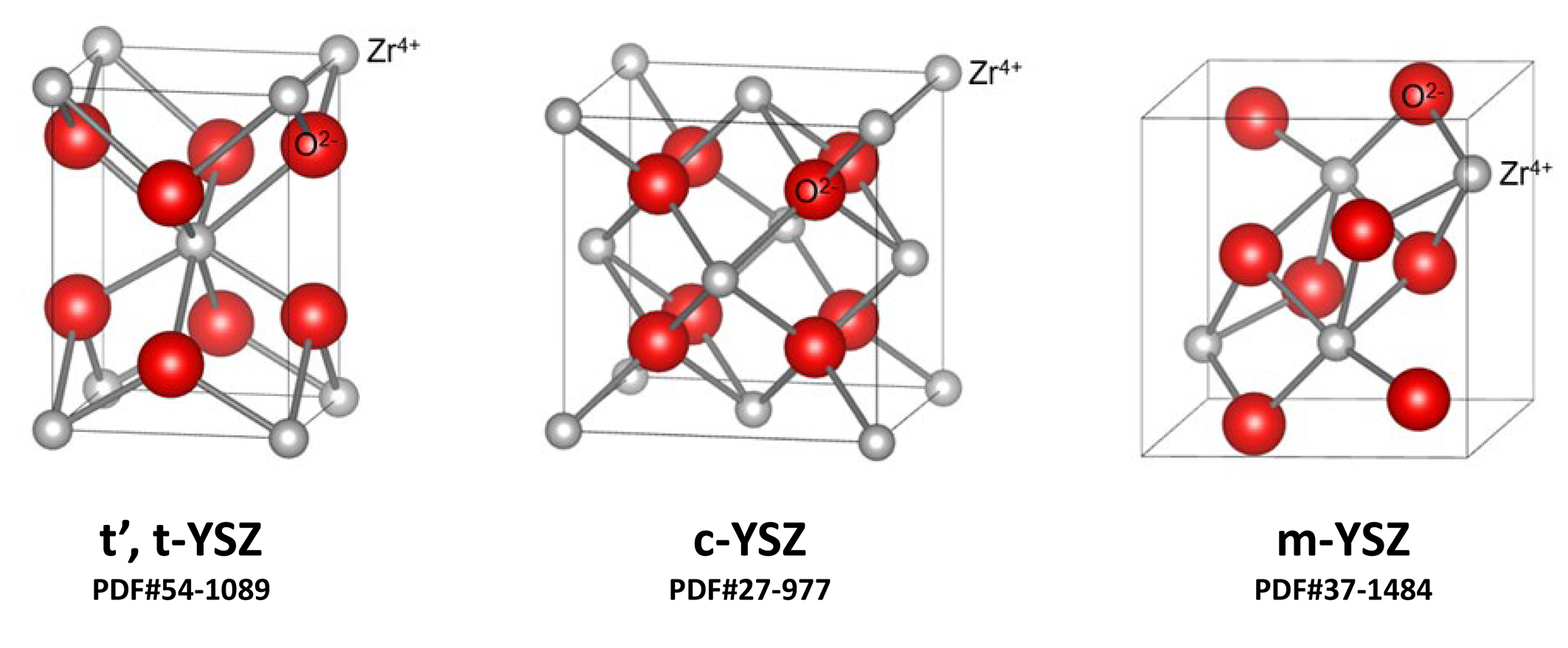

Supplement: Supplementary file 1 [file ijms-24-07663-s001.zip › Figure S4.tif]
